# Stromal CD10 expression in breast cancer correlates with tumor invasion and cancer stem cell phenotype

**DOI:** 10.1186/s12885-017-3951-8

**Published:** 2018-01-06

**Authors:** Tahani Louhichi, Hanene Saad, Myriam Ben Dhiab, Sonia Ziadi, Mounir Trimeche

**Affiliations:** Department of Pathology, Farhat-Hached University Hospital, 4000 Sousse, Tunisia

**Keywords:** Breast cancer, CD10, CD44, ALDH1, Cancer stem cell

## Abstract

**Background:**

Previous investigations have indicated that CD10 is associated with biological aggressivity in human cancers, but the use of this marker for diagnosis and prognosis is more complex. The aim of this study was to evaluate the expression of CD10 in breast cancer and its association with the clinicopathological features. In addition, we investigated whether a relationship exists between CD10 expression and cancer stem cells.

**Methods:**

CD10 expression was examined by the immunohistochemistry in a series of 133 invasive breast carcinoma cases. Results were correlated to several clinicopathological parameters. Cancer stem cell phenotype was assessed by the immunohistochemical analysis of CD44 and ALDH1.

**Results:**

Significant CD10 expression was found in the fusiform stromal cells in 19.5% of the cases and in the neoplastic cells in 7% of the cases. The stromal CD10 positivity was more frequently found in tumors with lymph node metastasis (*p* = 0.01) and a high histological grade (*p* = 0.01). However, CD10 expression by the neoplastic cells correlates with a high histological grade (*p* = 0.03) and the absence of estrogen (*p* = 0.002) as well as progesterone (*p* = 0.001) receptor expression.

We also found that CD10 expression by the stromal cells, but not by the neoplastic cells, correlates significantly with the expression of cancer stem cell markers (CD44+/ALDH1+) (*p* = 0.002).

**Conclusion:**

These findings support the role of the stromal CD10 expression in breast cancer progression and dissemination, and suggest a relationship with cancer stem cells.

## Background

Breast cancer represents the second most common cancer in the world [1]. Its prognosis remains poor because of metastasis and local recurrences, which are the main causes of therapeutic failure. This failure may be related to the cells’ nature composing tumor and to their biological characteristics.

Additional complexity is provided by the intervention of the tumor microenvironment that interacts with cancer cells and modifies several aspects of tumor development, including tumor growth, angiogenesis, invasion as well as metastatic dissemination. Adapting anti-cancer treatment to the tumor’ heterogeneity is an important challenge for future therapies.

Recently, the tumor microenvironment has also been considered as a potential therapeutic target because stromal cells are not as genetically unstable as cancer cells, and are therefore less likely to develop drug resistance [2]. A better understanding of tumor-stromal interactions could contribute to the identification of new therapeutic approaches that consider both stromal and tumor changes [3].

CD10 is a 90–110-kDa cell surface zinc-dependent metalloprotease that has been called common acute lymphoblastic leukemia antigen (CALLA) [4]. This enzyme is normally expressed by the epithelial cells of diverse tissues including prostate, colon, liver and stomach [5–7]. This protease is known to regulate the biological activities of various peptide substrates by lowering the local concentrations available for receptor binding [8].

Several reports have shown that the dysregulation of CD10 expression is significantly correlated with tumor progression and aggressiveness in a large variety of cancers such as melanoma [9], colorectal cancer [10] and nasopharyngeal cancer [11]. In breast cancer, many studies reported that the stromal CD10 expression is associated with more biologically aggressive tumors [12–14] and they have addressed the clinical significance of CD10 expression, however the use of CD10 for diagnosis and prognosis remain unclear [15]. On the other hand, few researches have investigated the significance of CD10 expression by the neoplastic breast cancer cells [16, 17]. More recent in-vitro studies have defined CD10 as a marker of stem-like or bipotent progenitor breast cells [18].

In the current study, we evaluated the expression of CD10 in a large series of breast cancer. We also assessed its relationship with several clinicopathological parameters including patients’ outcome and intrinsic molecular subgroups. In addition, we investigated whether a relationship exists between CD10 expression and breast cancer stem cell immunophenotype (CD44+/ALDH1+).

## Methods

### Patients and specimens

This study included 133 invasive ductal carcinomas, the most common breast cancer type, obtained from the archives of the Department of Pathology, Farhat Hached University Hospital of Sousse (Tunisia).

Different clinical and histological parameters including patient age, menopausal status, tumor size, lymph node metastasis, histological grade, hormone receptor and HER2 status, were collected from the Central Cancer Registry. Follow-up data were available for 74 patients. The overall follow-up time ranged from 4 to 143 months, with a median follow-up of 48 months, during which 11 patients underwent cancer relapse and 6 died.

The slides were reviewed and the cases were classified into 4 categories based on the immunohistochemical status of estrogen receptors (ER), progesterone receptors (PR), HER2 and Ki67, according to Goldhirsch et al. [19]: luminal A (ER+ and/or PR+, HER2−, low Ki67), luminal B (ER+ and/or PR+, HER2+ and/or high Ki67), HER2 overexpressing (ER−, PR−, HER2+) and triple negative (ER−, PR−, HER2−).

### Analysis of CD10 expression

The expression of CD10 (clone 56C6; dilution 1:100; Novocastra) was investigated by immunohistochemistry using the EnVision Flex system (DakoCytomation, Glostrup, Denmark) according to the manufacturer’s instructions.

Briefly, Paraffin-embedded breast cancer tissues was cut at 5 μm, dried overnight at 60 °C and deparaffinized in Ottix Plus (Diapath, Martinengo, Italy). Subsequently, the sections were hydrated with Ottix Shapper (Diapath, Martinengo, Italy), and rehydrated in water.

For antigen retrieval, the sections were boiled in a water bath with citrate buffer (0.01 M, pH 6.0) for 40 min until the temperature reached 98 °C. They were then allowed to cool at room temperature for 20 min, and placed in EnVision Flex Wash buffer (DakoCytomation, Glostrup, Denmark)**.** The endogenous peroxidase activity was blocked with EnVision Flex Peroxidase-Blocking Reagent for 5 min. The sections were thoroughly washed with the Wash buffer. The samples were incubated 30 min at temperature room with the primary antibody. Subsequently, the sections were rinsed gently with Wash buffer.

The sections were stained using the high sensitive polymer-based EnVision Flex/HRP system. After being rinsed in wash buffer, they were incubated in 3, 3 diaminobenzidine a substrate–chromogen solution for 20 min. Finally, the slides were counterstained with Mayer hematoxylin, permanently mounted, and viewed with a standard light microscope. Positive and negative controls were included for antibody according to the kit instructions.

The immunostaining results were evaluated independently by two pathologists (M.T. and S.Z.). CD10 expression was evaluated in the neoplastic and stromal cells. A case was considered positive if more than 10% of the cells exhibited positive signal, otherwise it was negative [11, 20].

### Analysis of cancer stem cell phenotype

The cancer stem cell phenotype was assessed by the immunohistochemical analysis of the expression of two cancer stem cell markers CD44 (clone DF1485, dilution 1:100, Leica, Newcastle, UK) and ALDH1 (clone 400 M-15, dilution 1:100, Cell Marque, Rocklin, California, USA). The antigen retrieval method and revelation system were the same as described for CD10.

Sequential double staining immunohistochemistry protocol was also used for CD10, ALDH1 and CD44 staining. We used the Bond Polymer Refine Red Detection (Fast Red) and the Bond Polymer Refine Detection (DAB) for the automated Bond system (Leica Microsystems, Wetzlar, Germany).

CD44 expression was evaluated in the cell membrane, whereas ALDH1 expression was evaluated in the cytoplasm of the tumor cells. For the two antibodies, a case was considered positive if more than 10% of the cells exhibited immunostaining, otherwise it was considered negative [10, 12, 21, 22].

### Statistical analysis

Data analysis has performed using the SPSS software (version 20.0; SPSS, Chicago, IL, USA). The correlation between CD10 expression and the clinicopathological parameters was investigated by the Chi-square and the Fisher exact tests, where appropriate. The survival analyses were performed according to the Kaplan-Meier method and compared by the log-rank test. A *p* value ≤0.05 was considered to indicate statistical significance.

## Results

### Features of CD10 expression in breast cancer

**Fig. 1:**
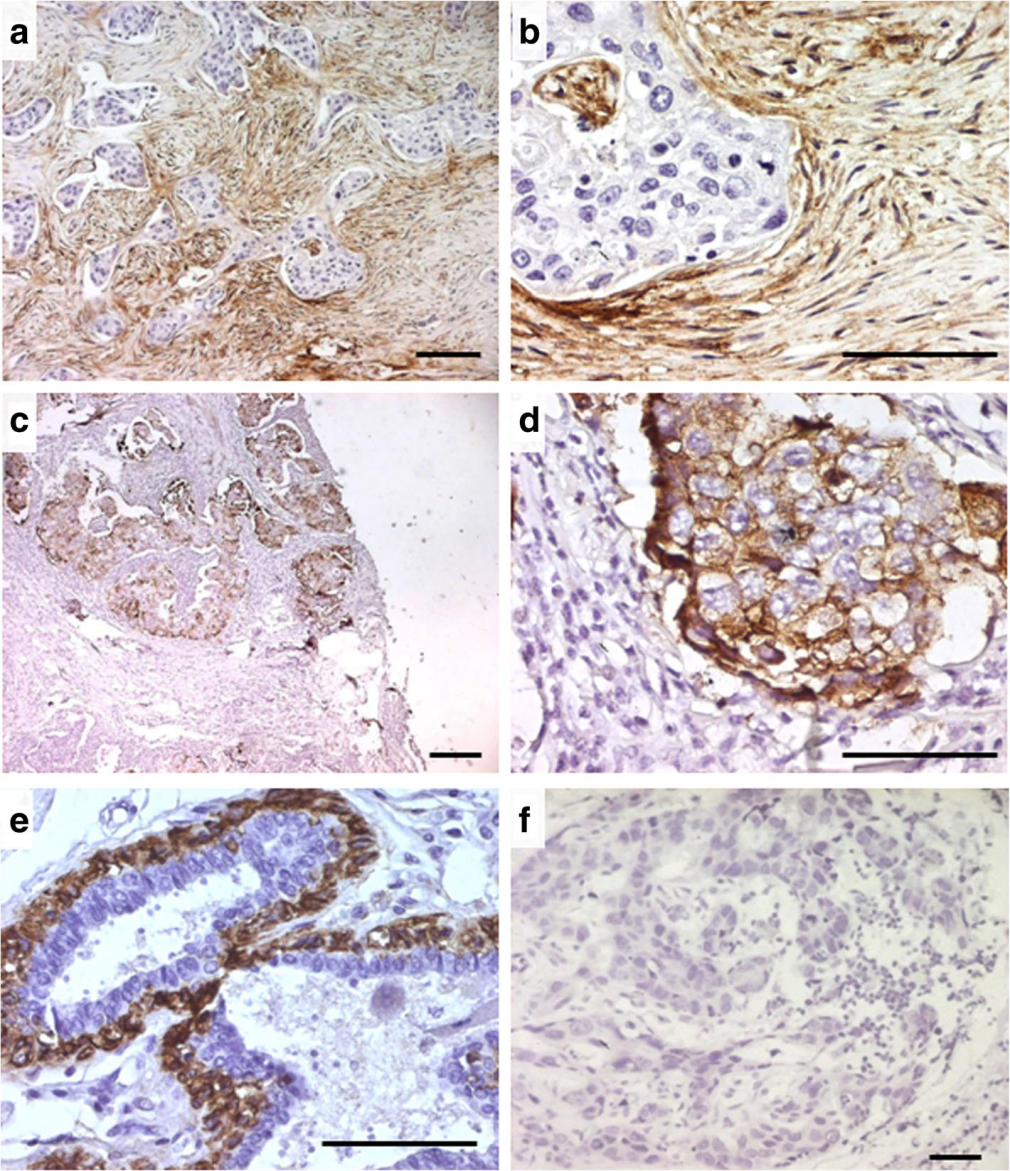
Patterns of CD10 expression in breast cancer. **a**, **b** strong CD10 positivity in the fusiform stromal cells, **c**, **d** in the neoplastic cells, and **e** in the normal myofibroblastic/basal layer cells of the adjacent non-tumoral acini and tubules. **f** negative control obtained by replacing the primary antibody by an universal IgG antibody (immunoperoxydase, scale bare = 0.1 mm, ×100 and ×400)

Twenty-six cases out of the 133 (19.5%) breast tumors investigated in this study showed a positive staining for CD10 in the stromal cells. In the positive cases, a strong signal was found in the cytoplasm of the stromal fusiform cells (Fig. 1a-b). Furthermore, a positive staining of CD10 was observed in the tumor cells in 9 cases (Fig. 1c-d), 3 among them showed concomitant staining for CD10 in the stromal cells. We also noted a positive staining for CD10 in the myoepithelial cell layer of the normal acini and tubules located in the breast tissue adjacent to the tumor in most cases (Fig. 1e). We also made an IHC negative control to show the staining specificity of CD10 (Fig. 1f).

### Correlation between CD10 expression and the clinicopathological parameters

The investigation of the correlations between CD10 expression and the clinicopathological parameters is shown in Table 1.

**Table 1:** Correlation between CD10 expression and clinicopathological parameters in breast cancer

Clinicopathological parameters	Total	CD10 expression in stromal cells		CD10 expression in tumoral cells	
		*n* (%)	*p*-value*	*n* (%)	*p*-value*
Age					
< 35 years	18	4 (22.2)	0.33	1 (5.5)	0.87
35–50 years	63	9 (14.2)		5 (7.9)	
> 50 years	52	13 (25)		3 (5.7)	
Menopausal status					
Pre-	70	15 (21.4)	0.56	6 (8.5)	0.49
Post-	63	11 (17.4)		3 (4.7)	
Familial history					
Positive	10	3 (30)	0.11	1 (10)	0.37
Negative	102	11 (10.7)		4 (3.9)	
Histological grade^a^					
Grade I	26	0 (0)	**0.01**	0 (0)	**0.03**
Grade II	56	12 (21,4)		1 (1.7)	
Grade III	51	14 (27.4)		8 (1.5)	
Tumor size					
≤ 20 mm	43	6 (13.9)	0.26	2 (4.6)	0.5
> 20 mm	90	20 (2)		7 (7.7)	
Lymph node metastasis^b^					
No metastasis	46	9 (19.5)	**0.01**	3 (6.5)	0.9
1 to 3 lymph nodes	37	3 (8.1)		3 (8.1)	
> 3 lymph nodes	20	8 (40)		1 (5)	
Visceral metastases					
Positive	10	0 (0)	0.35	0 (0)	1
Negative	78	14 (17.9)		5 (6.4)	
Estrogen receptors^c^					
Positive	62	11 (17.7)	0.62	1 (1.6)	**0.002**
Negative	71	15 (21.1)		8 (11.2)	
Progesterone receptors^c^					
Positive	65	10 (15.3)	0.23	1 (1.5)	**0.001**
Negative	68	16 (23.3)		8 (11.2)	
HER2 status^d^					
Positive	32	8 (25)	0.37	3 (9.3)	0.5
Negative	101	18 (17.8)		6 (5.9)	

^a^Scarff–Bloom and Richardson classification

^b^Thirty patients did not have lymph node resection

^c^Estrogen and progesterone receptors status was evaluated by immunohistochemistry, and considered positive if ≥1% of tumor cells showed nuclear staining

^d^Evaluated by immunohistochemistry and considered positive if scored 3+

*Bold number indicates significant *p*-value

We found that CD10 positivity in the stromal cells was more frequent in tumors with lymph node metastasis (*p* = 0.01) and in tumors with a high histological grade (*p* = 0.01).

We also found that CD10 positivity in the neoplastic cells correlate significantly with a high histological grade (*p* = 0.03). Moreover, an inverse correlation was found between CD10 positivity in the neoplastic cells and ER (*p* = 0.002) and PR (*p* = 0.001) expression.

**Fig. 2:**
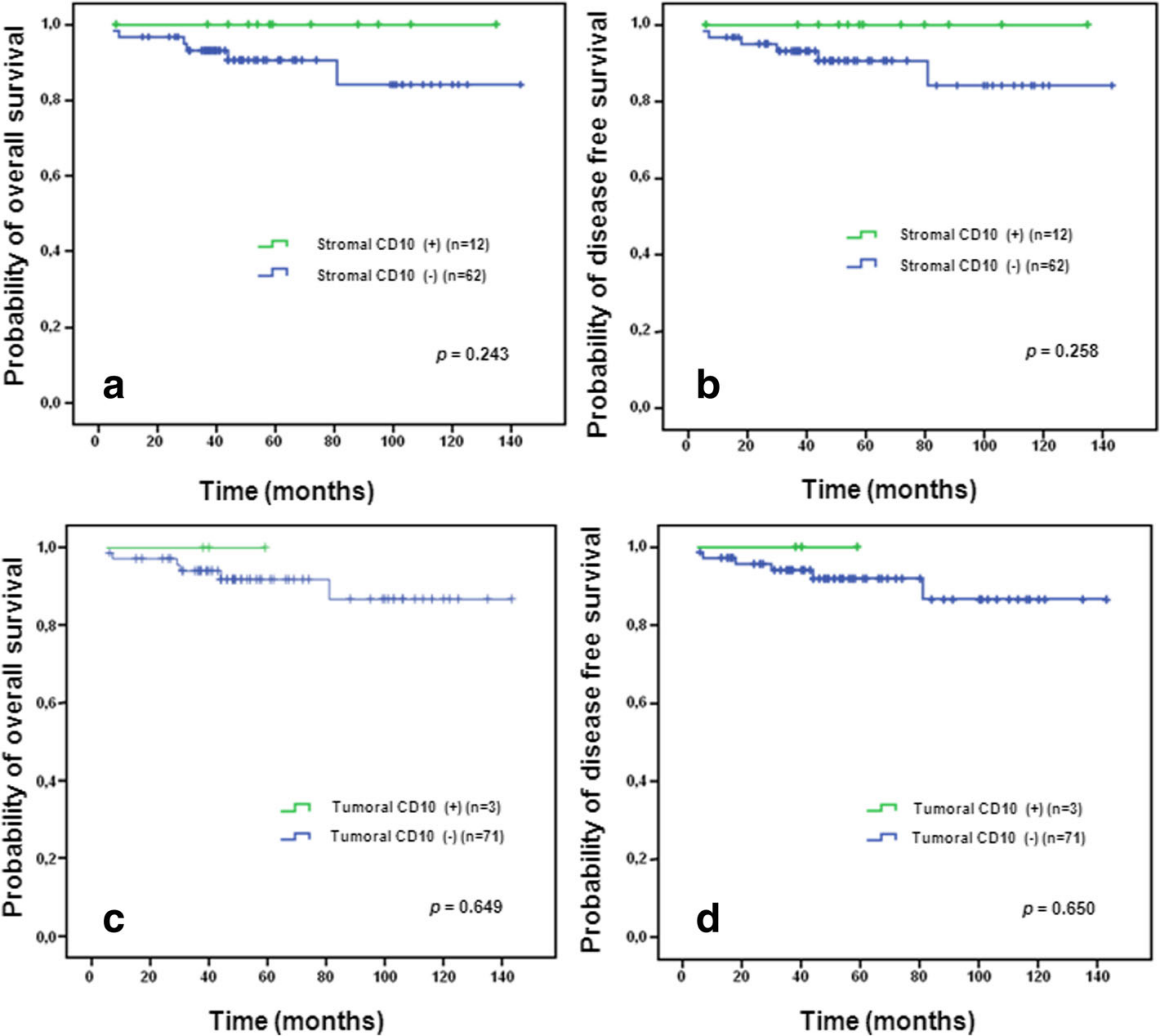
Kaplan-Meier curves of (**a**, **b**) overall survival and (**c**, **d**) disease-free survival according to CD10 expression status in patients’ with breast cancer. Follow-up data were only available for 74 patients in our series

The survival data analysis according to the status of CD10 expression in the stromal and the neoplastic cells showed no significant differences (Fig. 2).

### Association between CD10 expression and breast cancer molecular subgroups

Among the 133 breast cancer cases investigated, 64 (48.1%) were classified luminal A, 16 cases (12%) luminal B, 16 cases (12%) HER2 and 37 cases (27.9%) triple negative. The examination of the CD10 expression status according to these molecular subgroups (Table 2) showed that tumors with CD10 positivity in the neoplastic cells were more prevalent in triple negative (13.5%) and HER2 (18.7%) groups compared to luminal A (1.5%) and luminal B (0%) (*p* = 0.01). The differences do not reach the statistical significance for stromal CD10 expression.

**Table 2:** Correlation between CD10 expression and intrinsic molecular subtypes in breast cancer

Intrinsic molecular subtype	Total	CD10 expression in stromal cells		CD10 expression in tumoral cells	
		*n* (%)	*p*-value*	*n* (%)	*p*-value*
Luminal A	64	9 (14)	0.36	1 (1.5)	**0.01**
Luminal B	16	3 (18.7)		0 (0)	
HER2+	16	5 (31.2)		3 (18.7)	
Triple negative	37	9 (24.3)		5 (13.5)	

*Bold number indicates significant *p*-value

### Associations between CD10 expression and breast cancers stem cells

**Fig. 3:**
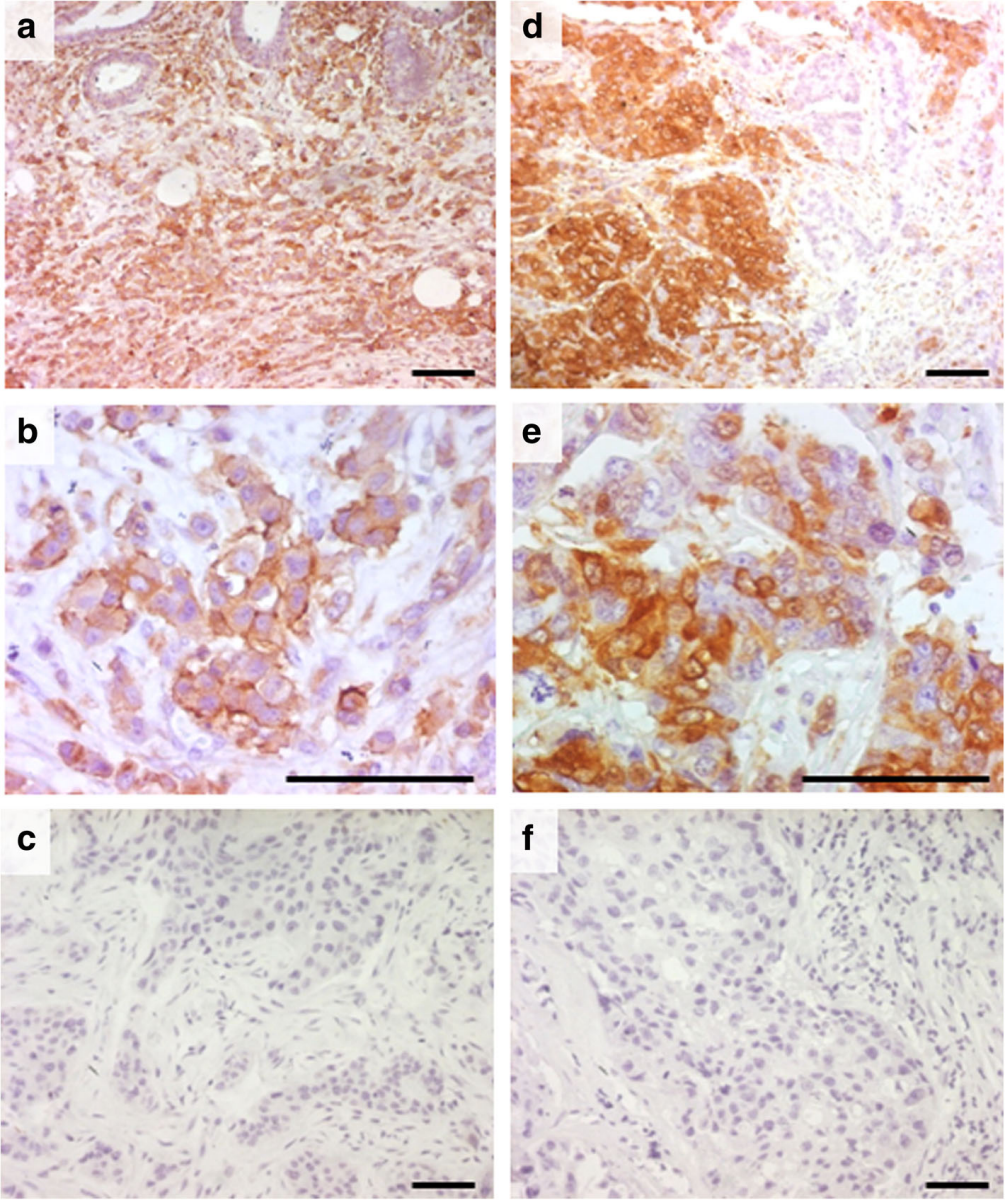
Examples of immunostaining for CD44 and ALDH1 in breast cancer. **a**, **b** strong membranous expression of CD44, and **d**, **e** cytoplasmic for ALDH1, in almost all the neoplastic cells. **c**, **f** negative control for CD44 and ALDH1 obtained by replacing the primary antibody by an universal IgG antibody (scale bare = 0.1 mm, ×100 and ×400)

The Cancer stem cell phonotype was assessed by the immunohistochemical analysis for the expression of two stem cell markers CD44 and ALDH1. We found that 42 of the 112 (30%) cases investigated express of at least one of the two stem cell markers. Indeed, 38 of the 112 (31%) breast cancer cases showed a strong membranous positivity for CD44 in most tumor cells (Fig. 3a-b). Yet, 10 of the 112 cases (9%) showed heterogeneous cytoplasmic expression of ALDH1 in the neoplastic cells (Fig. 3d-e). We also made an IHC negative control to show the staining specificity of CD44 and ALDH1 (Fig. 3c-f).

**Fig. 4:**
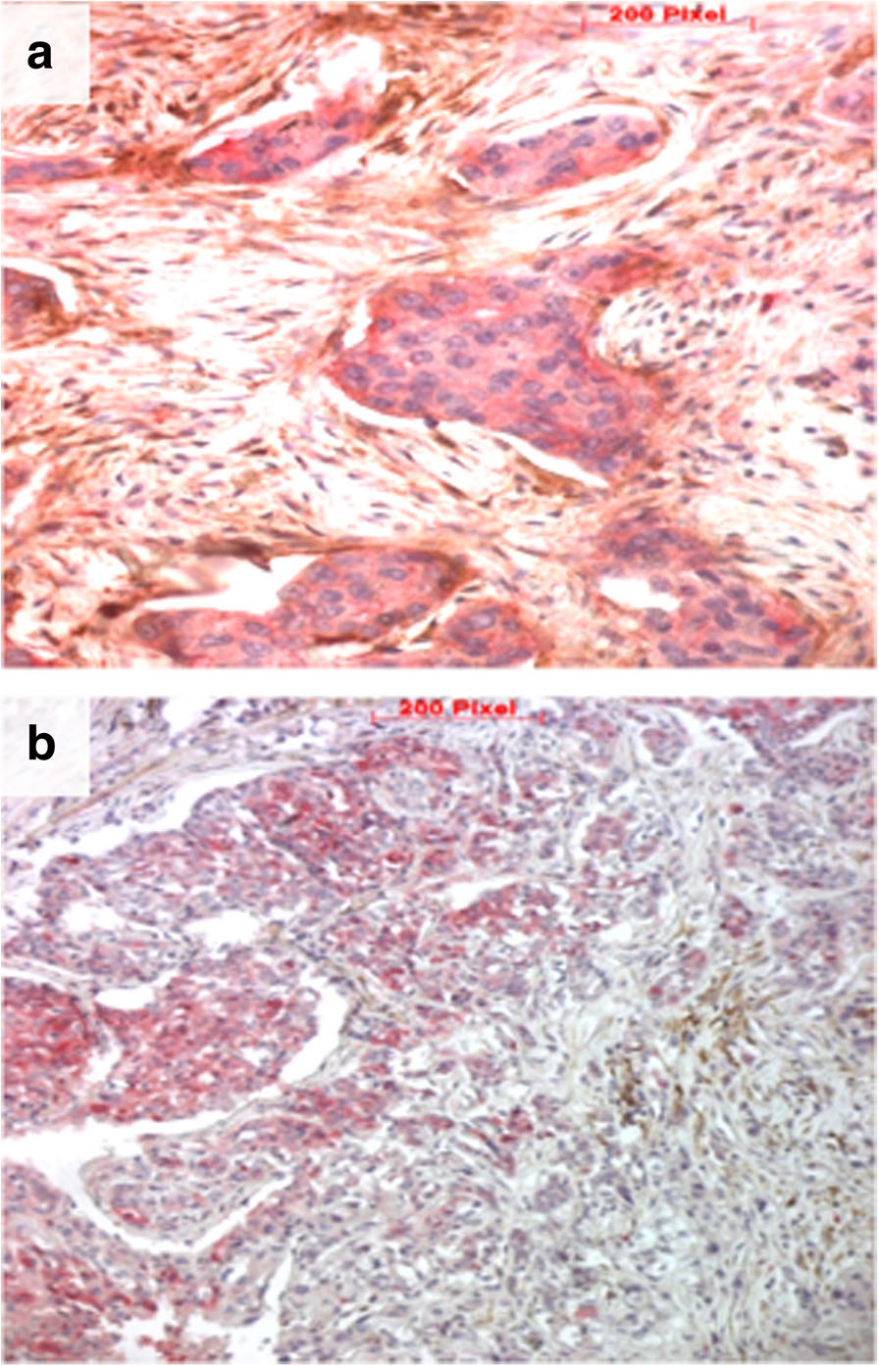
Double immunohistochemical staining (**a**) for CD44 (red staining) and CD10 (brown staining), and (**b**) for ALDH1 (red staining) and CD10 (brown staining) (scale bare = 0.1 mm, ×200)

The investigation of the relationship between CD10 and cancer stem cell markers (Table 3 and Fig. 4) showed that CD10 expression by the stromal cells was more frequent in tumors with cancer stem cell phenotype compared to tumors without this phenotype (*p* = 0.002). The difference remains significant when each of the two stem cell markers is analyzed separately (*p* = 0.01 and *p* = 0.008, respectively for CD44 and ALDH1). However, no significant correlation was found between CD10 positivity in the neoplastic cells and any of the cancer stem cell markers.

**Table 3:** Association between CD10 expression and breast cancer stem cell markers

Cancer stem cell markers	Total	CD10 expression in stromal cells		CD10 expression in tumoral cells	
		*n* (%)	*p*-value*	*n* (%)	*p*-value*
CD44 expression					
Negative	74	9 (12)	**0.01**	5 (7)	0.82
Positive	38	12 (31)		3 (8)	
ALDH1 expression					
Negative	102	16 (16)	**0.008**	7 (7)	0.70
Positive	10	5 (50)		1 (10)	
CD44 and ALDH1			**0.002**		0.48
Negative	106	2 (2)		6 (6)	
Positive	6	4 (67)		0 (0)	

*Bold numbers indicate significant correlations

## Discussion

Recently, it is well documented that the interaction of cancer cells with their microenvironment promotes tumor progression [23]. This interaction involves several factors that influence signaling pathways related to tumor invasion and metastatic dissemination. Understanding the interaction between cancer cells and stromal cells in the tumor microenvironment may be useful for screening potential candidate markers and new therapeutic targets [24].

In the present study, we analyzed the expression of CD10 in the stromal and neoplastic cells through a series of breast cancer to assess whether CD10 is associated with a particular clinicopathological feature. We showed that CD10 is expressed by the fibroblastic stromal cells and the neoplastic epthelial cells, respectively in 19.5% (26 of 133) and 7% (9 of 133) of the cases. Previous researches have investigated CD10 expression in breast cancer and they have reported positivity’s variable rates, ranging from 18 to 53.4% [13, 14, 17] and 6 to 7% [25, 26], respectively in the stromal and the neoplastic cells. Several hypotheses have been advanced to explain this variability in the reported rates, including the heterogeneity in the tested series, and the differences in the methods and cut-off values used in these studies [13, 16, 17].

With regard to the clinicopathological parameters, we found a significant association between the stromal CD10 positivity, a high histological grade (*p* = 0.01) and the presence of lymph node metastasis (*p* = 0.01). These findings are in agreement with several previous reports [14, 17, 27] which suggest a role of CD10 in the local invasion and metastatic dissemination of the neoplastic cells.

Acquisition of an invasive and metastatic character is accompanied by the mammary tumor cells’ ability to secrete substances such as matrix metallo-proteases (MMP). MMPs are involved in the degradation of the matrix extracellular’ proteins, leading neoplastic cells to leave the primary lesion and to invade at distant tissues [27, 28]. As like other matrix metalloproteases family, CD10 expression by the stromal cells may contribute to the tumor progression.

In this regard, previous reports had reported that CD10 is strongly expressed by stromal cells in the advanced stages of some cancers, including breast cancer, colorectal cancer, melanoma but not in primary tumors stage [8, 10, 13].

A recent gene expression profiling study identified two types of stromal signatures in breast cancer, namely, solitary fibrous tumor type and desmoid-type fibromatosis. According to this study, the first type was correlated with a poor prognosis and was associated with the expression of CD10 [29].

Further studies have shown that CD10 positive stroma signature includes genes involved in matrix remodeling (MMP11, MMP13, and COL10A1) [29–32]. Interestingly, this signature showed an important role in differentiating in situ from invasive breast cancer, proving the fact that progression from in situ to invasive breast cancer is dependent upon the tumor microenvironment.

In the current study, 9 of the 133 (7%) breast cancer cases showed a positive staining for CD10 in the neoplastic cells. All these cases exhibit A high histological grade (*p* = 0.03). This finding indicates that CD10 expression in the neoplastic cells also reflects the aggressive character of the tumor. Moreover, we found that CD10 expression by the neoplastic cells was significantly associated with hormonal receptor negativity (*p* = 0.002 and *p* = 0.001, respectively for ER and PR). When we investigated whether an association exists between CD10 and the molecular subtypes of breast cancer, we found that CD10 positivity in the neoplastic cells was more frequent in triple negative (13.5%, 5/37 cases) and HER2 (18.7%, 3/16 cases) tumors compared to luminal A and B tumors (1.5%, 1/80 cases, *p* = 0.01). This finding is consistent with an earlier work by Livasy et al. [25] exploring CD10 expression in three subgroups of breast cancer using DNA microarray analysis (18 basal-like, 12 HER2+ and 16 luminal). The authors found a CD10 expression by the neoplastic cells in 12.5% ​(2/16 cases) of the basal-like group against only 3.6% (1/28 cases) for the other groups, suggesting that CD10 positive breast cancers arise from basal/myoepithelial cells which normally do not express hormone receptors.

In the current study, we were also interested in seeing how CD10 can influence the tumor microenvironment and can favor tumor extension and dissemination. Indeed, this protease appears to be an important regulator for the breast tissue [33]. Indeed, expressed on the surface of epithelial or stromal cells, CD10 degrades the extracellular matrix through its enzymatic activity, thus facilitating the propagation of the neoplastic cells and their dissemination. The tumor microenvironment, consisting of adjacent supporting cells and molecules of the extracellular matrix, is required to maintain the identity of the stem cells and therefore their ability to self-renew [34]. It plays an essential role in regulating the cancer stem cells’ behavior. In this context, we investigated the relationship between CD10 and cancer stem cell phenotype. For this purpose, we evaluated the expression of two cancer stem cell markers CD44 and ALDH1. We found that the stromal expression of CD10 was more frequent in tumors with cancer stem cell phenotype compared to tumors without this phenotype (*p* = 0.002). This significant correlation persists even when we analyze each of the two stem cell markers separately (*p* = 0.01 and *p* = 0.008, respectively for CD44 and ALDH1). However, no correlation was found between CD10 positivity in the neoplastic cells and these markers. The former finding indicates that tumors with stem cell phenotype are closely related to the stromal CD10 expression and thus to the aggressiveness and invasiveness of breast cancers. To our knowledge, our study is the first report that addresses the relationship between CD10 expression and cancer stem cell markers in breast cancer.

Our findings indicated that breast tumors with cancer stem cells phenotype may induce the expression of CD10 in the tumor microenvironment. Acting as a co-receptor for many growth factors and cytokines, including the metalloproteases produced by cells in the tumor microenvironment, CD44 could mediate their signaling preferentially transduced into CD44-positive tumor cells to stimulate cancer stem cells self-renewal and promote invasion and metastasis [35]. Indeed, this will favor the destruction of the extracellular matrix and consequently the propagation of the neoplastic cells that will join the lymphatic system and give distant metastases.

The analysis of our patients’ outcome according to the CD10 expression status in the stromal or the neoplastic cells did not reveal any significant difference in overall or relapse-free survival. This could be due to the limited number of cases for which we have follow-up data or to some confusing factors. Only few studies have evaluated the impact of CD10 expression on the clinical outcome of breast cancer patients. Two of them have found a significant association between the clinical outcome and CD10 expression [13, 17], suggesting that CD10 expression may serve as a prognostic factor in the same manner as other previous biomarkers in breast cancer, combining with hormonal, HER2 status and Ki67 routinely. However, large-scale multicenter studies are needed to clarify this issue.

## Conclusions

In summary, we analyzed the expression of CD10 in a large series of breast cancers. We found that CD10 is significantly expressed by the fusiform stromal cells in 19.5% of the cases, and in the neoplastic cells in 7% of the cases. We also found that breast tumors expressing CD10 were significantly associated with the clinical parameters of aggressiveness and invasiveness, including a high histological grade and the presence of nodal metastasis. We also showed a significant association between the stromal CD10 positivity and tumors with cancer stem cell phenotype (expressing CD44 and ALDH1). These findings support a role of CD10, as a metalloprotease, in the progression and dissemination of breast cancer, and suggest the implication of the cancer stem cells in the induction of CD10 expression by the stromal cells. Further studies are required to elucidate the mechanism by which the cancer stem cells induce a stromal CD10 expression and to evaluate its utility as a target to develop new therapies.

## Abbreviations

CALLA: Common acute lymphoblastic leukemia antigen
ER: Estrogen receptors
IHC: immunohistochemistry
PgR: Progesterone receptors
SBRs: Scarff-Bloom-Richardson system
